# Some Like It Cold: Long‐Term Assessment of a Near‐Global Invader

**DOI:** 10.1002/ece3.70760

**Published:** 2024-12-22

**Authors:** S. Guareschi, T. Cancellario, F. J. Oficialdegui, A. Laini, M. Clavero

**Affiliations:** ^1^ Department of Life Sciences and Systems Biology University of Torino Torino Italy; ^2^ Centre Balear de Biodiversitat Universitat de les Illes Balears Palma de Mallorca Spain; ^3^ Faculty of Fisheries and Protection of Waters, South Bohemian Research Center of Aquaculture and Biodiversity of Hydrocenoses University of South Bohemia in České Budějovice Vodňany Czech Republic; ^4^ Department of Conservation Biology and Global Change Estación Biológica de Doñana, EBD‐CSIC Sevilla Spain

**Keywords:** climatic suitability, global change, invasion trajectory, invasive species, invertebrates, long‐term data

## Abstract

Long‐term studies depicting the multicontinental invasion trajectories of species are often constrained by the scarcity of documented records, especially for invertebrates. The red swamp crayfish, *Procambarus clarkii* (Decapoda: Cambaridae), stands out as an uncommon example of hypersuccessful invasive species with a well‐known invasion history at both regional and global levels. This allows for the use of its records to track distribution dynamics and bioclimatic preferences over time. Through multiple temporal comparisons, the global bioclimatic tendencies of the species have been explored over a period exceeding a century (1854–2023) using linear models with generalized least squares estimation and two‐sample t‐tests. This specific setup provides a rare focus on biological invasions at both broad temporal and spatial scales. The results highlight climatic trends in the invasion process of the species, including decreases in the values of bioclimatic variables associated with temperature and precipitation. This trend encompasses not only mean values but also both extreme (minimum and maximum) and is coupled with increases in elevation and aridity values in the areas with the presence of the species. The findings indicate that the species can engage in new ecological interactions and further affect range‐restricted species in climatic refuges once considered protected. These findings help anticipate changes in the species' invasion trajectory, suggesting possible expansions into colder, less humid climates and higher altitudes. This knowledge supports effective monitoring and early detection for management and conservation efforts.

## Graphical abstract

Through multiple temporal comparisons, global bioclimatic tendencies of the species have been explored over more than a century (1854–2023), representing a rare focus at both wide temporal and spatial scales. The findings indicate that the species can engage in new ecological interactions and further affect range‐restricted species in climatic refuges once considered protected. These findings help anticipate changes in the species' invasion trajectory, suggesting possible expansions into colder, less humid climates and higher altitudes.

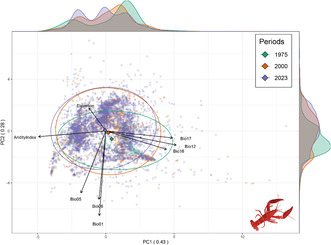

## 1 Introduction

Biological invasions are a challenging component of global change that yields diverse and multiple impacts (IPBES 2023). However, they also indirectly provide opportunities to explore spatiotemporal processes and ecological theories (Leprieur et al. 2008; Guareschi et al. 2022; Juozaitienė et al. 2023). For instance, “niche conservatism hypothesis” (i.e., the tendency of species‐climate associations to remain constant over space and time) triggered debate when dealing with biological invasions (e.g., Bates and Bertelsmeier 2021). Liu et al. (2020) advocated that most invasive species largely occupy similar climatic niches in their native and introduced ranges, whereas Hill, Gallardo, and Terblanche (2017) found that niche expansion is common among invasive invertebrates. Indeed, climate has an important role on species distributions, especially for ectotherms (Barbet‐Massin et al. 2018; Gallardo, Zieritz, and Aldridge 2015). At the same time, high‐resolution climatic data have become increasingly available on a global scale over time (e.g., Karger et al. 2017), opening up new research opportunities when combined with long‐term species occurrence data.

Nevertheless, long‐term research has traditionally been challenging in biodiversity conservation and invasion science, primarily due to the scarcity of biological data, such as geo‐localized occurrences (e.g., Rick and Lockwood 2013). Comparisons between native and invaded areas, involving a space–time substitution, remain the predominant approach in biological invasions (e.g., Lustenhouwer and Parker 2022). Moreover, the exploration of bioclimatic trends (i.e., long‐term tendencies and assessment of preferences) for invasive species, at both wide spatial and temporal scale, is particularly rare. In this context, common focuses at small temporal and spatial scales risk hindering the usefulness of invasion research to managers (e.g., Matzek, Pujalet, and Cresci 2015).

Crayfish are traditionally valued in human societies for their diverse uses and have been widely transported, ranking among the most widespread and impactful non‐native species in freshwater ecosystems (e.g., Lodge et al. 2012). Among them, the red swamp crayfish, *Procambarus clarkii* (Girard, 1852) (Decapoda: Cambaridae) represents an extensively and historically studied species, whose expansion trajectory is well known at regional and global levels (Larson and Olden 2012; Loureiro et al. 2015; Oficialdegui et al. 2019; Guareschi et al. 2024). The species is native to Southern United States and North‐eastern Mexico and has been introduced in other areas since the 1920s. Its subsequent near‐global expansion has been linked to the growth of the aquaculture industry, and more recently, to pet trade, being currently present in numerous countries on all continents except Antarctica and Australasia (Oficialdegui, Sánchez, and Clavero 2020). The spread of this polytrophic species has been associated with detrimental ecological effects on multiple ecosystem elements, including amphibians, insects, submerged vegetation, and waterfowl birds (Rodríguez et al. 2005; Manenti et al. 2020; Watanabe and Ohba 2022) as well as economic implications and spread of pathogens (Souty‐Grosset et al. 2016; Kouba et al. 2022).

As a well‐documented aquatic species extensively monitored both within and beyond its native regions, with reliable records from the 19th century, *P. clarkii* provides a unique opportunity to use distributional data for long‐term bioclimatic assessments (Ion et al. 2024). Recently, findings from Guareschi et al. (2024) supported a bioclimatic niche stability of the species but also evidenced some bioclimatic expansions during its multicontinental invasion trajectory. However, the entity and nature of these changes have not been studied and defined so far. By focusing on multiple well‐defined variables, we aim to unravel long‐term bioclimatic trends in the species' distribution over more than a century via multiple temporal comparisons. This approach aids in better understanding the species' spread and enables more accurate predictions of future distribution shifts, thereby informing targeted management strategies and conservation efforts.

## 2 Materials and Methods

### 2.1 Biological and Bioclimatic Data

Occurrences of red swamp crayfish from 1854 to 2023 were gathered from the most comprehensive bibliography (Oficialdegui, Sánchez, and Clavero 2020; Guareschi et al. 2024), which compiled information from scientific and gray literature, technical reports, and online platforms. This resulted in a total of > 54,000 occurrences at a global scale for both native and invaded areas.

**FIGURE 1:**
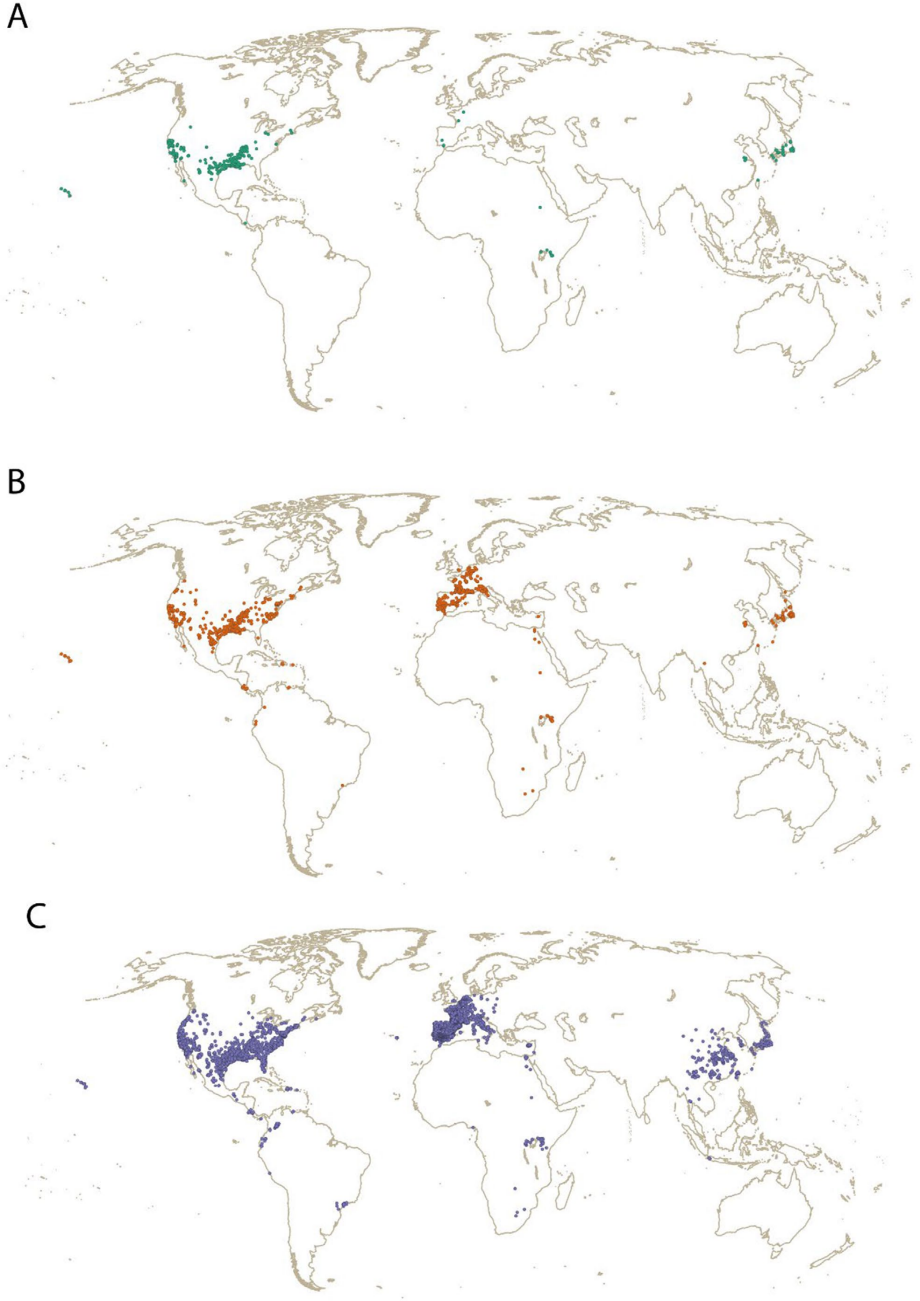
Occurrences used in statistical analysis after the spatial thinning procedure. Approach with three groups: Up to 1975 (A, green); up to 2000 (B, dark orange); and up to 2023 (C, violet). Maps showing the approach with two groups are available in Figure S1. Records from Oficialdegui, Sánchez, and Clavero (2020) and Guareschi et al. (2024), see details in the Methods section.

To perform a long‐term bioclimatic assessment, we grouped and compared the occurrences in two different and complementary ways. In the first approach, we split the occurrences into three periods of time (Figure 1) aligning with the expansion trajectory of *P. clarkii* : (i) up to 1975, (ii) up to 2000, and (iii) up to 2023 (see Oficialdegui, Sánchez, and Clavero 2020). In the second approach, we divided the dataset into two large groups: (i) all occurrences up to 2000 and (ii) more recent occurrences from 2001 to 2023 (see Figure S1). While the first approach may have nested data (e.g., the last period has data from mid‐19th century to 2023), the second approach ensures a focus on two clearly separated periods of time, without the possibility of having the same record in both groups.

To minimize potential spatial biases during statistical analysis we applied a spatial thinning approach using the R package “dismo” (Hijmans et al. 2023). This approach ensures that just one record for each raster cell (10 arcmin: ~18 × 18 km at equator) was considered from each period. After reducing spatial correlation and eliminating duplicate records, the approach of three groups guarantees 342, 923, and 4709 records, respectively, while 923 (up to 2000) and 4320 (for the period 2001–2023) in the second case (Figures 1 and S1).

Climatic data were obtained from CHELSAcruts (first period up to 1975) and CHELSA V2.1 datasets (recent periods after 1975) (Karger et al. 2017; Karger and Zimmermann 2018). To have a complete overview we focused on a pool of eight biologically relevant climatic variables able to represent the bioclimatic space of the species in terms of extremes and mean values. Three variables were temperature‐related: annual mean value (BIO01) and both extremes (BIO05 = max temperature of the warmest month; BIO06 = min temperature of the coldest month). Three variables were related to precipitation: annual mean value (BIO12) and both extreme values (BIO16 = precipitation of wettest quarter; BIO17 = precipitation of driest quarter). This list was further complemented with the Thornthwaite Aridity Index (hereinafter Aridity Index) obtained using the R package “envirem” (Title and Bemmels 2018) and Elevation (cell mean altitude above sea level) obtained from WorldClim v. 2.1 (Fick and Hijmans 2017). Similar variables have been used to define climate niche in large‐scale assessments of invertebrates facilitating comparisons across taxa (e.g., Hill, Gallardo, and Terblanche 2017).

To climatically characterize each period, we averaged climatic variables over the previous 10 years (e.g., for the first period: we averaged values from 1966 to 1975), except for the last period, where we averaged climatic data from 2009 to 2018. A complete list of variables and definitions is available in Table S2.

### 2.2 Statistical Analysis

We assessed bioclimatic differences among the three groups of occurrences (up to 1975, up to 2000, and up to 2023) using a linear model with generalized least squares (gls) (R package “nlme,” Pinheiro and Bates 2023). Due to the unbalanced nature of the datasets, we ran two models for each bioclimatic variable: a standard one [gls(predictor ~ Period)] and one allowing for unequal variances among periods [gls(predictor ~ Period, weights = varIdent(form = ~ 1 | Period))]. We then proceeded with the model displaying the lowest AIC (Akaike Information Criterion) value (see Table S3). Post hoc pairwise comparisons between periods were then performed using estimated marginal means, and *p*‐values were adjusted for multiple comparisons via Tukey method within the “emmeans” package, which also provides effect size values (Lenth 2024). Models were then validated by checking the graphical distribution of residuals (log‐transformed, if necessary, i.e., Elevation) and data plotted with the “ggplot2” package (Wickham 2016).

When we focused on just two periods (before and after 2000), we compared the bioclimatic variable using two‐sample *t*‐test. Cohen's *d* was calculated as effect size measures using the package “effectsize” (Ben‐Shachar, Lüdecke, and Makowski 2020). The ratio of variances for each variable was explored and being close to 1 (i.e., 0.90–1.35), heterogeneity was excluded (Blanca et al. 2018). The normality of each variable was graphically checked, and data log‐transformed when necessary.

For both approaches (dataset split into three or two periods), sites with the presence of the species were ordered using a principal component analysis (PCA) based on the eight bioclimatic variables considered in the modeling to summarize the invasion trajectory of the species across time. All statistical analyses were performed in the R environment (R v. 4.3.2; R Core Team 2024).

## 3 Results

**FIGURE 2:**
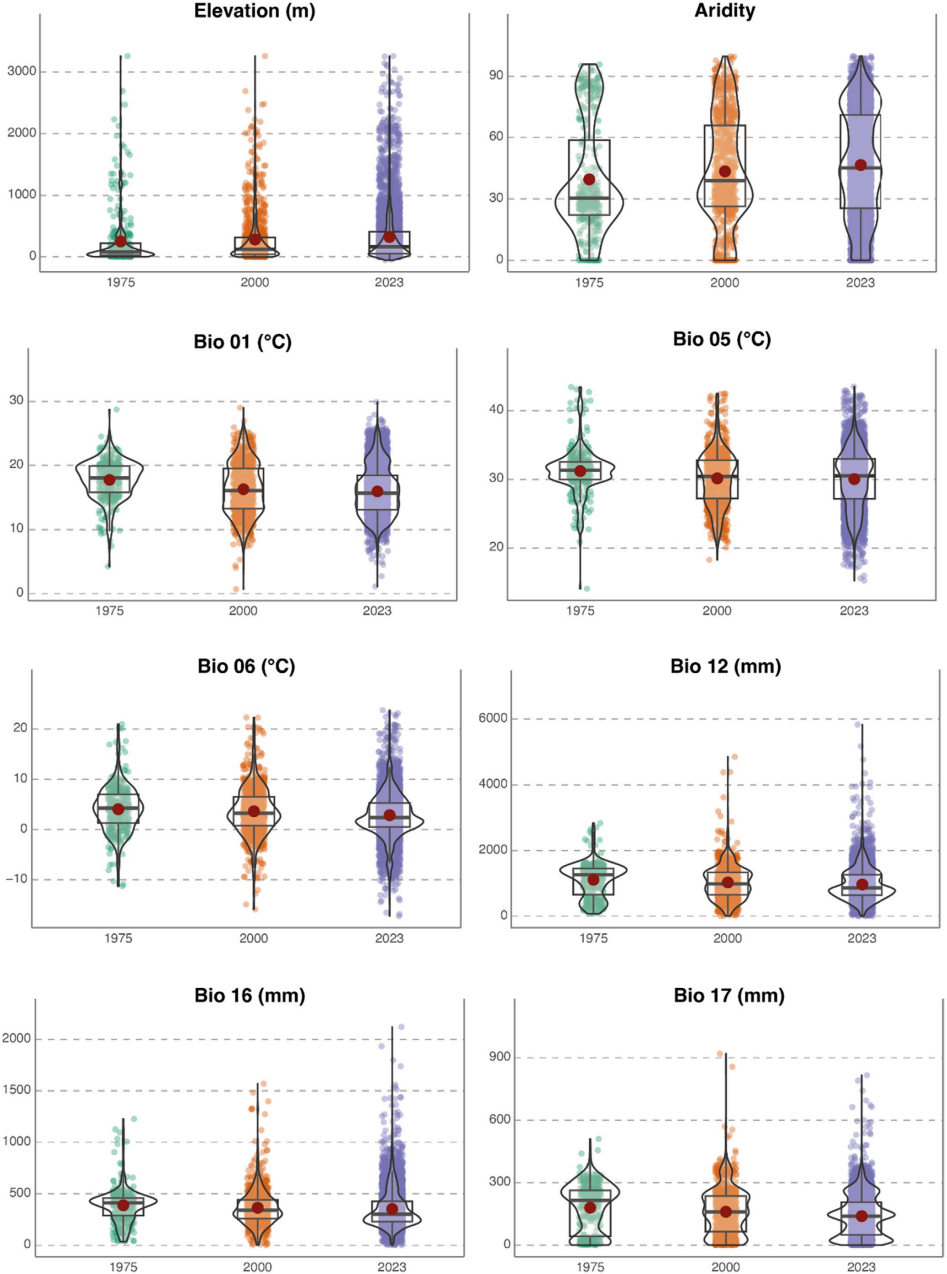
Violin plots with the density of values for each of the variables from 3 periods of time (up to 1975, up to 2000, and up to 2023). Box covers the interquartile interval; the bold horizontal line represents the median while the red dot is the mean value. Aridity is expressed as Thornthwaite aridity index (the higher the value, the greater the aridity). Statistics, *p*‐values, and effect size values are available in Table 3.

**FIGURE 3:**
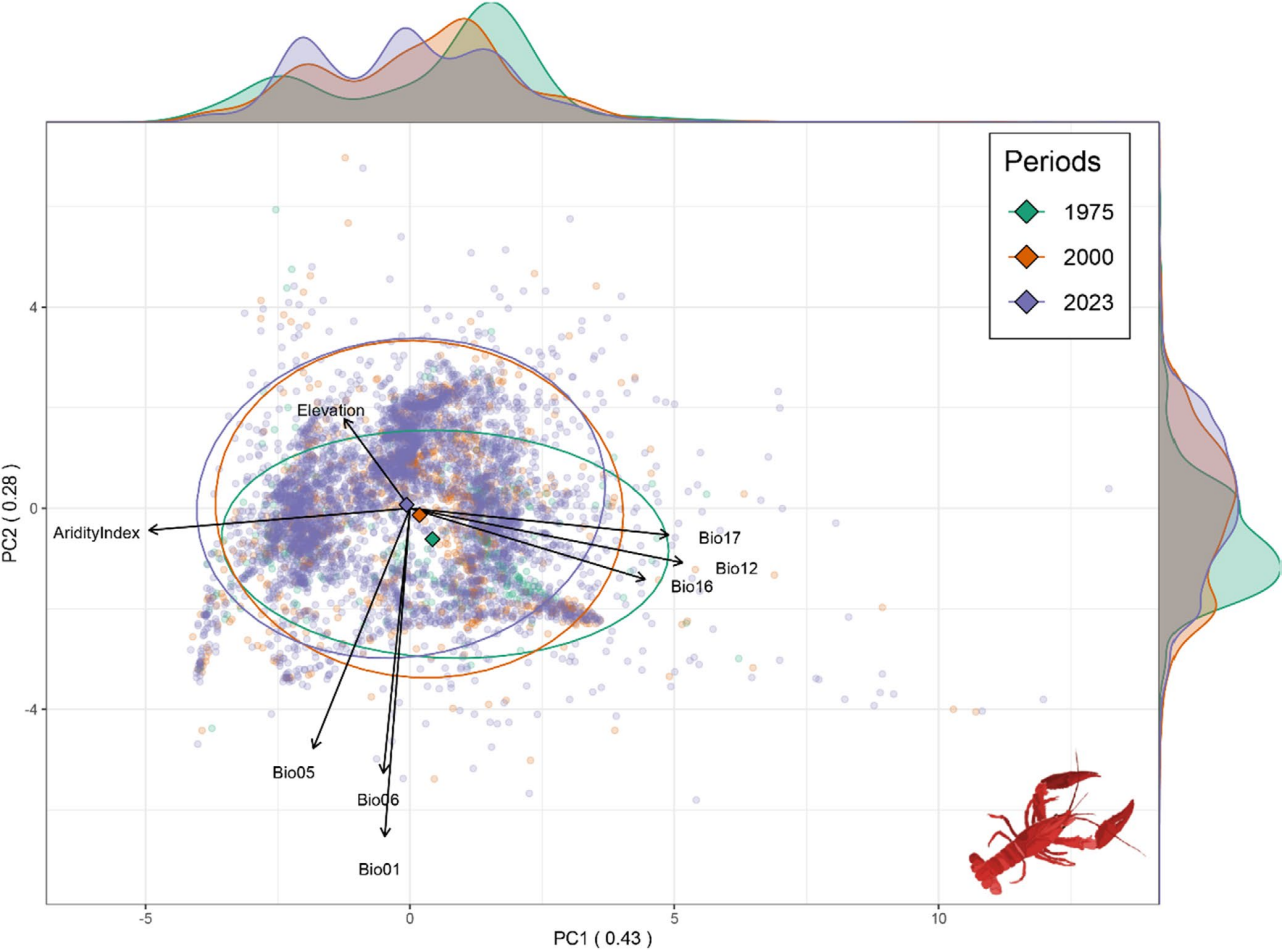
Principal components analysis with the variables used when modeling (definitions available in the main text and Table S2). Two axes (PC1 and PC2) are displayed. 95% confidence ellipses and centroids for the three periods are also displayed in green (up to 1975), orange (up to 2000), and purple (up to 2023). Marginal plots show distributions for each period in both axes.

The study of the near‐global invasion trajectory of *P. clarkii* revealed some bioclimatic patterns in its long‐term distribution (Figures 1, 2, 3). In the first approach (three periods), moving from accumulated records up to 1975 to the more recent distribution (2023), descriptors of temperature and precipitation significantly tend toward lower values (*p*‐values < 0.001, details in Figure 2, Tables 1 and 2). The opposite tendencies can be appreciated for Elevation and Aridity Index values (both in mean and median: Figure 2, Tables 1 and 2). For example, focusing on annual mean temperature (BIO01), records up to 1975 showed annual average values of 17.8°C while the pool of records up to 2023 of 16.0°C (Table 1). Similarly, comparing the same periods, the annual mean precipitation (BIO12) exhibited a decrease in the mean and median values of approximately 140 and 400 mm, respectively (details in Table 1).

**TABLE 1:** Mean, standard deviation, and median values of variables (definitions available in the main text and Table S2) in areas with *P. clarkii* presence when considering three periods of time (up to 1975, up to 2000, and up to 2023).

Variable	Period	Mean	SD	Median
Elevation (m)	1975	249.8	455.7	79.0
	2000	282.4	426.0	122.0
	2023	323.1	438.7	164.0
Aridity index	1975	39.5	27.3	30.3
	2000	43.4	26.0	38.9
	2023	46.5	25.9	45.1
BIO01	1975	17.8	3.0	18.1
	2000	16.3	4.0	16.1
	2023	16.0	3.8	15.7
BIO05	1975	31.2	3.4	31.3
	2000	30.1	4.0	30.4
	2023	30.0	4.2	30.5
BIO06	1975	4.0	4.6	4.2
	2000	3.6	4.8	3.2
	2023	2.9	4.4	2.4
BIO12	1975	1108.0	520.4	1266.5
	2000	1028.4	523.7	984.1
	2023	967.5	488.9	861.2
BIO16	1975	388.9	165.8	412.1
	2000	365.1	176.0	341.8
	2023	352.7	187	303.2
BIO17	1975	179.7	114.9	215.7
	2000	160.3	111.9	159.8
	2023	139.3	99.2	139.5

*Note:* Values when using two groups are available in Table S4.

**TABLE 2:** Results of the generalized least squares (gls) models to assess the relevance of the variable “Period” (“1975,” “2000,” “2023”) for each bioclimatic variable.

Variable	numDF	*F*‐value	*p*	Model approach	Variation
Elevation	2	24.0	0.0001	gls	+
Aridity index	2	15.5	0.0001	gls	+
BIO01	2	56.0	0.0001	gls unequal var	−
BIO05	2	18.4	0.0001	gls unequal var	−
BIO06	2	19.3	0.0001	gls unequal var	−
BIO12	2	15.7	0.0001	gls unequal var	−
BIO16	2	8.5	0.0001	gls unequal var	−
BIO17	2	31.5	0.0001	gls unequal var	−

*Note:* Variation column can be (+) in case of increase or (−) in case of decrease according to mean and median values of Table 1. Elevation data were log‐transformed. numDF = numerator degrees of freedom. Definitions available in Table S2 (and in the main text).

Generalized least squares allowing for unequal variances among groups performed better (in terms of lower AIC) for most of the variables, with the only exceptions of Elevation and Aridity Index (complete details in Table S3). For all variables, the factor “Period” was detected as significant (Table 2). Effect sizes were highly variable (ranging from 0.07 to 0.60) but tended to be higher when comparisons involved the most distant periods (e.g., 1975 vs. 2023, Table 3).

**TABLE 3:** Results of the pairwise comparisons (Tukey adjustments) and effect size analysis considering three periods of time (“1975,” “2000,” “2023”) and their combinations.

	Period	Estimate	*t*‐ratio	*p*	Effect size
Elevation	1975–2000	−0.2	−3.14	0.0049	−0.198
	1975–2023	−0.3	−6.08	0.0001	−0.340
	2000–2023	−0.1	−3.94	0.0002	−0.142
Aridity index	1975–2000	−3.9	−2.37	0.047	−0.150
	1975–2023	−7.0	−4.81	0.0001	−0.269
	2000–2023	−3.1	−3.31	0.0027	−0.119
BIO01	1975–2000	1.5	7.02	0.0001	0.489
	1975–2023	1.8	10.53	0.0001	0.601
	2000–2023	0.3	2.37	0.047	0.113
BIO05	1975–2000	1.1	4.70	0.0001	0.313
	1975–2023	1.2	6.06	0.0001	0.345
	2000–2023	0.1	0.77	0.723	0.033
BIO06	1975–2000	0.42	1.43	0.329	0.092
	1975–2023	1.19	4.67	0.0001	0.261
	2000–2023	0.77	4.51	0.0001	0.169
BIO12	1975–2000	79.6	2.41	0.043	0.153
	1975–2023	140.5	4.84	0.0001	0.270
	2000–2023	61.0	3.27	0.003	0.117
BIO16	1975–2000	23.8	2.23	0.067	0.144
	1975–2023	36.2	3.86	0.0004	0.218
	2000–2023	12.3	1.93	0.1315	0.074
BIO17	1975–2000	19.5	2.70	0.019	0.169
	1975–2023	40.4	6.33	0.0001	0.352
	2000–2023	20.9	5.29	0.0001	0.182

The first PCA axis explained 43% of the bioclimatic variance representing a precipitation gradient (BIO12, BIO16, BIO17, and Aridity Index) while the second axis accounted for 28% of the variance and was related to temperature variables (Figure 3). The centroids of each distribution were relatively close together, but data distribution showed a shift in both axis over time toward colder and drier areas (Figure 3).

**FIGURE 4:**
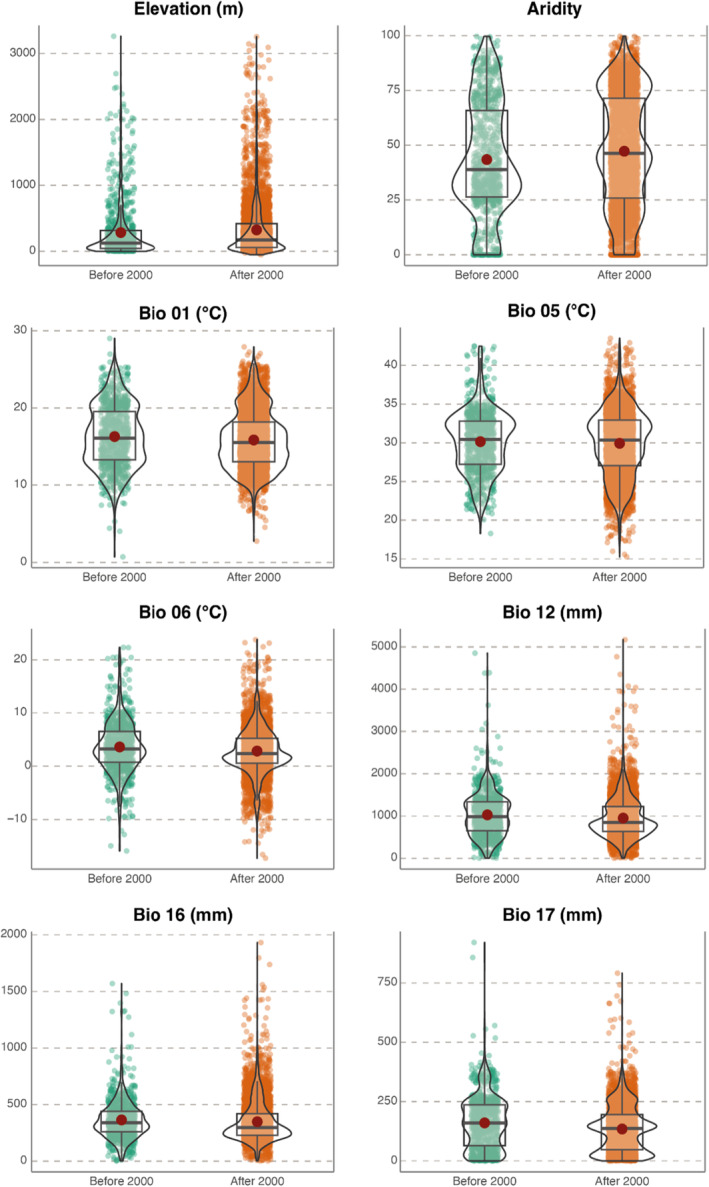
Violin plots with the density of values for each of the variables from 2 periods of time (≤ 2000 and > 2000). Box covers the interquartile interval; the bold horizontal line represents the median while the red dot is the mean value. Aridity is expressed as Thornthwaite aridity index. Statistics, *p*‐values, and effect size values are available in Table S5.

When grouping the occurrences of the species on two exclusive periods (presence up to 2000 and in the period 2001–2023), the same tendencies observed in the 3‐period analysis can be detected with a reduction in temperature and precipitation and increases in elevation and aridity values (Figure 4, Tables S4 and S5). For example, an elevational shift of at least 40 m in the average altitude values can be observed when comparing occurrences before and after the year 2000 (282.4 m a.s.l. vs. 322.7 m a.s.l., Table S4). Effect sizes ranged from 0.05 to 0.26 (details in Table S5) while graphical PCA representation showed similar patterns to those of the previous three groups‐approach (available in Figure S6).

## 4 Discussion

The ability and success of *Procambarus clarkii* in establishing populations across a wide range of environments have been associated with its high ecological plasticity (Loureiro et al. 2015). This adaptive capacity can hinder the disentanglement of the species' bioclimatic space, especially when focusing on limited spatial and temporal data. In this study, we explored the multicontinental invasion trajectory of *P. clarkii* over more than a century of time, representing a rare example of focus at both wide temporal and spatial scales.

Our findings revealed climatic tendencies in the long‐term invasion process of *P. clarkii* , including a trend toward occupying colder and dryer areas. This not only involved mean values but also both maximum and minimum data. These results are concordant with the shift toward higher altitudes and, up to a certain point, also with the increases in the Aridity Index values detected. Nevertheless, they also emphasized contrasts with the humid subtropical climate found in the species' native regions, such as southern United States (e.g., Louisiana). Elevation trends are probably driven by the recent expansion of the species into mountainous regions (e.g., South America: Oficialdegui, Sánchez, and Clavero 2020; De Oliveira et al. 2023), while, at the same time, some previously colonized warm sites are experiencing longer and more pronounced dry periods (e.g., Mediterranean region: Drobinski et al. 2020). The burrowing capacity of the species, which is also emphasized in drought contexts and fluctuating environmental conditions (Kouba et al. 2016) may aid in adaptation and it is likely to broaden its ecological implications through water turbidity and habitat modification (e.g., Angeler et al. 2001; Neculae et al. 2024). Interestingly, while being recognized as a warm‐water species (Zhang et al. 2020), *P. clarkii* exhibits higher environmental plasticity than other invasive crayfish species (e.g., Viana et al. 2023). Its tolerance to lower temperatures (e.g., winter temperature simulations of European temperate zone: Veselý et al. 2015) and higher altitude regions (e.g., established populations in Colombia and recent records in pre‐alpine areas of Italy), coupled with scenarios of global change, could facilitate the arrival and successful spread of the species to new, yet uncolonized areas. Interestingly, the genomic adaptive potential of the species to cold environments has recently been emphasized in populations from cold‐climate areas of Japan (Sato et al. 2023). Rapid adaptive trait changes have been noted for numerous successful invasive species (e.g., fish and salinity: *Neogobius melanostomus* , Green, Havenhand, and Kvarnemo 2020, bivalves and temperature: *Corbicula fluminea* , Guareschi and Wood 2020). Nevertheless, recent emphasis has been placed on the substantial variability in spreading speed and abundance trends across biogeographic regions among non‐native freshwater invertebrates, both within and between species (Haubrock et al. 2024).

However, some aspects of climate change (e.g., altered water bodies hydroperiod and precipitation regimes) may not necessarily benefit the expansion of the species in all contexts and spatial scales. At global scale, focusing on future climatic scenarios and conservative climatic niche, Zhang et al. (2020) anticipated that regions with suitable climate for *P. clarkii* would expand in Europe but contract in North America, Asia, and some areas of the Mediterranean basin (see also Capinha, Anastácio, and Tenedório 2012, for an example focused on the Iberian Peninsula). The multiple and dynamic interactions between climate change and biological invasions would benefit from further research, in the case of *P. clarkii* of specific interest in both native and invaded areas, as well as in “contact regions” predicted as suitable areas but not invaded yet.

The trends stressed here are, in most of the cases, associated with moderate power in terms of effect size. A commonly used interpretation is to refer to effect sizes as small (*d* ≤ 0.2) and large (*d* ≥ 0.8) based on thresholds by Cohen (1988). Our findings stressed effect sizes < 0.2 in the approach of the two periods (except 0.26 for BIO17) while values mostly remained below 0.35 in the other approach, except for those observed in annual mean temperature (BIO01), particularly in the larger temporal window (1975 vs. 2023). This last trend appears to be linked to temporal proximity, as closer periods display a more uniform distribution due to shared base records and limited geographic expansion compared to more distant periods.

Notably, while mean and median values for temperature and precipitation values of areas with *P. clarkii* presence decreased over time, this trend was accompanied by a more comprehensive exploitation of the range of values in the latest time‐period for most variables (e.g., annual mean precipitation—BIO12 values in the three‐period approach). Similarly, when examining the most recent occurrences, a wide range of values emerges for annual mean temperature (BIO01), spanning from 0°C to 30°C. These findings support the bioclimatic plasticity of the species, which is particularly evident within the ranges and types of variables analyzed.

As a whole, the exploration of such a large dataset, both in spatial and temporal dimensions, allowed us to identify bioclimatic trends in the invasion trajectory of *P. clarkii* . The results, from multiple temporal comparisons, help anticipate future changes in the species' invasion trajectory warning for further expansions primarily toward colder, less humid climates, as well as higher mean altitudes. While Guareschi et al. (2024) have recently demonstrated some degree of global niche conservatism to effectively predict the expansion of the species, our new findings indicate slight shifts toward colder and drier areas upon further refinement. This aligns with the notion that niche in the invasive range tend to be more heterogeneous than in their native range (Barbet‐Massin et al. 2018), and it suggests that *P. clarkii* 's fundamental niche is likely much broader than its current realized niche, with a niche dynamic in space and time (e.g., Pearman et al. 2008).

In a scenario of global change, these shifts could lead to previously unexplored interactions within ecosystems and local or introduced species as well as impacts on range‐restricted species located in climatic refuges (e.g., mountainous areas). These regions, that often also include designated nature conservation areas, were historically considered protected by environmental filtering (e.g., headwater refuges and flow implications on certain non‐native crayfish species: Satmari et al. 2023). However, less lotic systems (e.g., lakes, reservoirs, or artificial waterbodies in high‐altitude areas) may be still vulnerable to targeted human‐mediated introductions, which could serve as sinks of new populations into these evolving bioclimatic contexts, thereby acting as “stepping stone” mechanism for further spread. Overall, this research offers a comprehensive exploration of the invasion trajectory of a highly successful species, providing valuable understanding of long‐term biological invasions. These insights hold significant applied importance for management and nature conservation, particularly in prevention and early detection efforts.

## Author Contributions

**S. Guareschi:** conceptualization (lead), data curation (equal), formal analysis (equal), investigation (equal), methodology (equal), software (equal), visualization (equal), writing – original draft (lead), writing – review and editing (equal). **T. Cancellario:** conceptualization (lead), data curation (equal), formal analysis (equal), investigation (equal), methodology (equal), software (equal), visualization (equal), writing – original draft (lead), writing – review and editing (equal). **F. J. Oficialdegui:** data curation (equal), investigation (equal), resources (equal), writing – review and editing (equal). **A. Laini:** formal analysis (equal), investigation (equal), methodology (equal), resources (equal), writing – review and editing (equal). **M. Clavero:** funding acquisition (equal), project administration (equal), writing – review and editing (equal).

## Conflicts of Interest

The authors declare no conflicts of interest.

## Supporting information


Data S1.

## Data Availability

The raw data that support the findings of this study are openly available in the platform Open Science Framework (OSF) at https://osf.io/j459a/?view_only=5d70555133124cec86fc837b802a7ced.
