# Supplementary material for: Some Like It Cold: Long‐Term Assessment of a Near‐Global Invader
Source: Ecol Evol. 2024 Dec 22;14(12):e70760. doi: 10.1002/ece3.70760 (PMC11663627; doi:10.1002/ece3.70760)

**SUPPLEMENTARY MATERIAL**

**Figure S1.** Occurrences used in statistical analysis after the spatial thinning procedure, with the approach considering two groups: before 2000 (A - green); after 2000 (B - dark orange).

**Table S2.** List and definitions of the bioclimatic variables considered in the research. (source and details in the main text).

**Table S3.** AIC (Akaike Information Criterion) values for each variable analysed and model approach used (gls and gls with unequal variance). Degrees of freedom (df) are also included. Definitions are available in S2.

**Table S4.** Mean, Standard Deviation, Median values for each variable considering 2 periods of time. Before and included 2000 (n. 943 occurrences) and after the year 2000 (n. 4,320 occurrences).

**Table S5.** Results of the t-test analysis comparing two groups of records (before and after the year 2000). Elevation data are log-transformed. Effect size values are displayed as Cohen's *d*.

**Figure S6.** Principal Components Analysis with the variables used when modelling (definitions available in the main text and S2). Two axes (PC1 and PC2) are displayed. 95% confidence ellipses and centroids for the two periods are also displayed. Marginal plots show distributions for each period in both axes.

**Figure S1.** Occurrences used in statistical analysis after the spatial thinning procedure, with the approach considering two groups: before 2000 (A - green); after 2000 (B - dark orange).


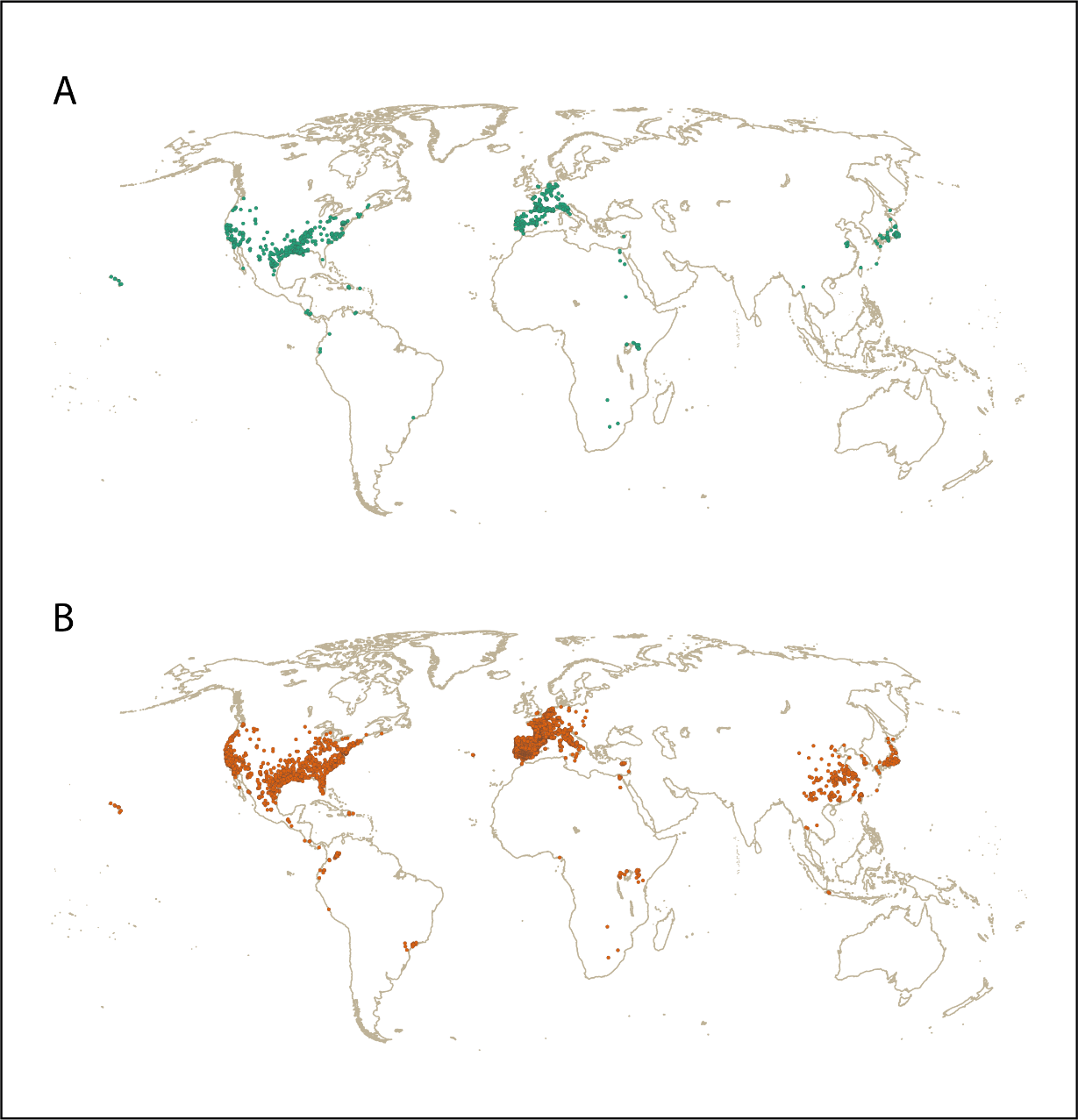


**Table S2.** List and definitions of the bioclimatic variables considered in the research (details in the main text).

| **Bioclimatic variables** | **Definition and Units** | **Source** |
| --- | --- | --- |
| BIO1 | Annual Mean Temperature (ºC) | WorldClim V2.1 |
| BIO5 | Max Temperature of Warmest Month (ºC) | WorldClim V2.1 |
| BIO6 | Min Temperature of Coldest Month (ºC) | WorldClim V2.1 |
| BIO12 | Annual Precipitation (mm) | WorldClim V2.1 |
| BIO16 | Precipitation of Wettest Quarter (mm) | WorldClim V2.1 |
| BIO17 | Precipitation of Driest Quarter (mm) | WorldClim V2.1 |
| Elevation | Altitude above sea level (mean value at cell scale) (m) | WorldClim V2.1 |
| AridityIndex | Thornthwaite aridity index: Index of the degree of water deficit below water need (-) | R package envirem |

**Table S3.** AIC (Akaike Information Criterion) values for each variable analysed and model approach used (gls and gls with unequal variance). Degrees of freedom (df) are also included. Definitions are available in S2.

| **Variables** | **Model approach** | **df** | **AIC** |
| --- | --- | --- | --- |
| Elevation | gls | 4 | 16241.3 |
|  | gls unequal var | 6 | 16244.5 |
| Aridity Index | gls | 4 | 55879.4 |
|  | gls unequal var | 6 | 55881.5 |
| BIO01 | gls | 4 | 32779.5 |
|  | gls unequal var | 6 | 32746.6 |
| BIO05 | gls | 4 | 33852.4 |
|  | gls unequal var | 6 | 33827.7 |
| BIO06 | gls | 4 | 34863.0 |
|  | gls unequal var | 6 | 34852.7 |
| BIO12 | gls | 4 | 91098.7 |
|  | gls unequal var | 6 | 91093.5 |
| BIO16 | gls | 4 | 79263.7 |
|  | gls unequal var | 6 | 79254.8 |
| BIO17 | gls | 4 | 72231.9 |
|  | gls unequal var | 6 | 72201.6 |

**Table S4.** Mean, Standard Deviation, Median values for each variable considering 2 periods of time. Before and included 2000 (n. 943 occurrences) and after the year 2000 (n. 4,320 occurrences).

| **Variable** | **Period** | **Mean** | **SD** | **Median** |
| --- | --- | --- | --- | --- |
| **Elevation (m)** | ≤ 2000 | 282.4 | 426.0 | 122 |
|  | > 2000 | 322.7 | 430.1 | 170 |
| **Aridity Index** | ≤ 2000 | 43.4 | 26.0 | 38.9 |
|  | > 2000 | 47.2 | 25.7 | 46.3 |
| **Bio01** | ≤ 2000 | 16.3 | 4.0 | 16.1 |
|  | > 2000 | 15.9 | 3.7 | 15.5 |
| **Bio05** | ≤ 2000 | 30.1 | 4.0 | 30.4 |
|  | > 2000 | 29.9 | 4.2 | 30.3 |
| **Bio06** | ≤ 2000 | 3.6 | 4.8 | 3.2 |
|  | > 2000 | 2.9 | 4.3 | 2.4 |
| **Bio12** | ≤ 2000 | 1028.4 | 523.7 | 984.1 |
|  | > 2000 | 950.9 | 476.1 | 846.8 |
| **Bio16** | ≤ 2000 | 365.1 | 176.0 | 341.8 |
|  | > 2000 | 348.9 | 184.7 | 297.8 |
| **Bio17** | ≤ 2000 | 160.3 | 111.9 | 159.8 |
|  | > 2000 | 134.6 | 96.3 | 137.5 |

**Table S5.** Results of the t-test analysis comparing two groups of records (before and after the year 2000). Elevation data are log-transformed. Effect size values are displayed as Cohen's *d*.

| **Variable** | **df** | **t-value** | **p-value** | **Variation** | **eff size** |
| --- | --- | --- | --- | --- | --- |
| Elevation | 5241 | -4.19 | 2.83e-05 | + | -0.15 |
| Aridity Index | 5241 | -4.09 | 4.40e-05 | + | -0.15 |
| BIO01 | 5241 | 3.25 | 1.17e-03 | - | 0.12 |
| BIO05 | 5241 | 1.45 | 0.1478 | - | 0.05 |
| BIO06 | 5241 | 4.85 | 1.27e-06 | - | 0.18 |
| BIO12 | 5241 | 4.41 | 1.05e-05 | - | 0.16 |
| BIO16 | 5241 | 2.43 | 1.50e-02 | - | 0.09 |
| BIO17 | 5241 | 7.14 | 1.05e-12 | - | 0.26 |

**Figure S6.** Principal Components Analysis with the variables used when modelling (definitions available in the main text and S2). Two axes (PC1 and PC2) are displayed. 95% confidence ellipses and centroids for the two periods are also displayed. Marginal plots show distributions for each period.


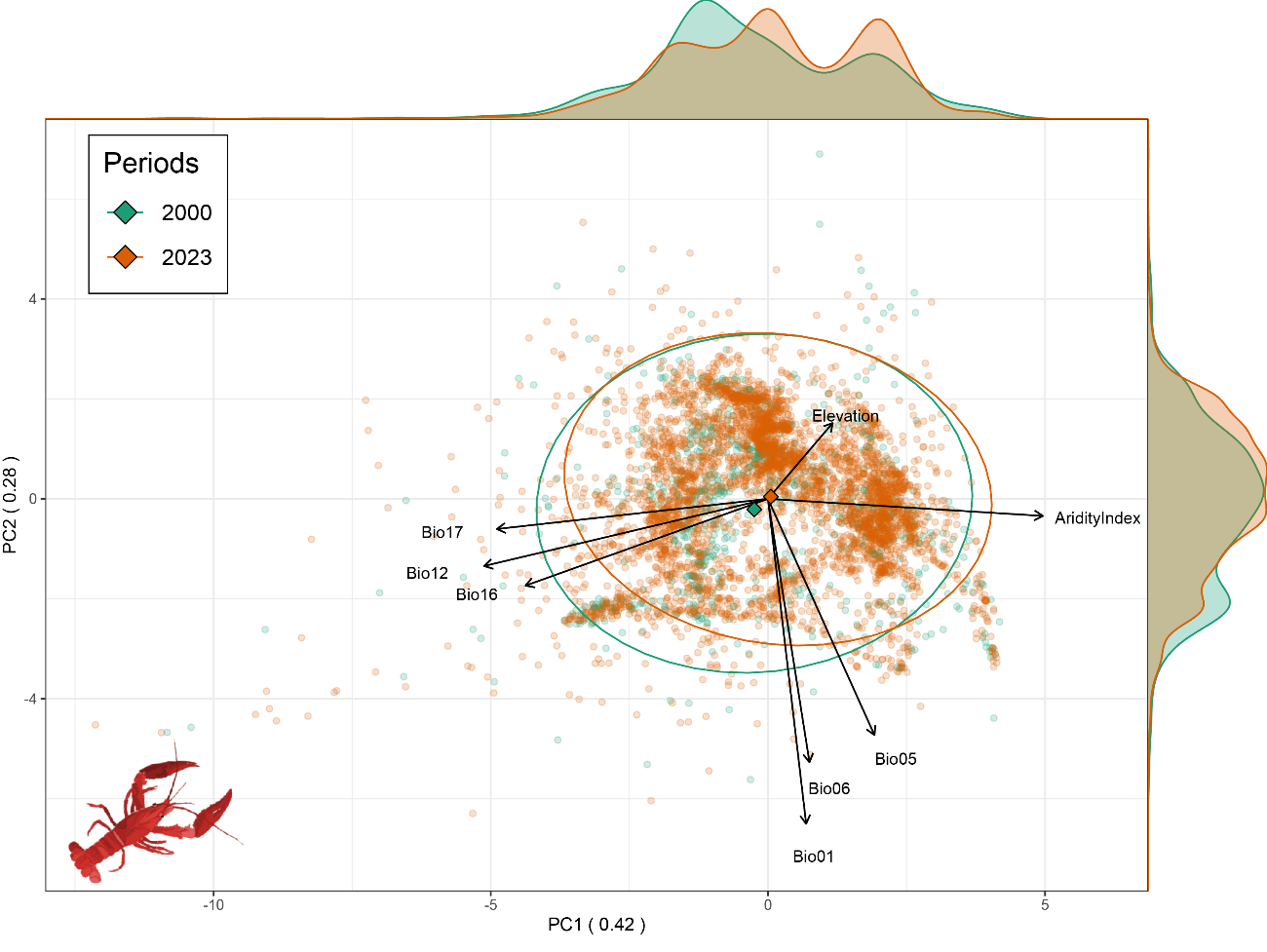

Supplement: Supplementary file 1 — Data S1. [file ECE3-14-e70760-s001.docx]
